# Benefits of Sealed-Curing on Compressive Strength of Fly Ash-Based Geopolymers

**DOI:** 10.3390/ma9070598

**Published:** 2016-07-20

**Authors:** Sujeong Lee, Arie van Riessen, Chul-Min Chon

**Affiliations:** 1 Korea Institute of Geoscience and Mineral Resources, Daejeon 34132, Korea; crystal2@kigam.re.kr (S.L.); femini@kigam.re.kr (C.-M.C.); 2 Department of Resources Recycling Engineering, University of Science and Technology, Daejeon 34113, Korea; 3 John de Laeter Centre, Curtin University, Perth 6845, Western Australia, Australia

**Keywords:** geopolymers, curing regime, strength development

## Abstract

There is no standardized procedure for producing geopolymers; therefore, many researchers develop their own procedures for mixing and curing to achieve good workability and strength development. The curing scheme adopted is important in achieving maximum performance of resultant geopolymers. In this study, we evaluated the impact of sealed and unsealed curing on mechanical strength of geopolymers. Fly ash-based geopolymers cured in sealed and unsealed moulds clearly revealed that retention of water during curing resulted in superior strength development. The average compressive strength of sealed-cured geopolymers measured after 1 day of curing was a modest 50 MPa, while after 7 day curing the average compressive strength increased to 120~135 MPa. In the unsealed specimens the average compressive strength of geopolymers was lower; ranging from 60 to 90 MPa with a slight increase as the curing period increased. Microcracking caused by dehydration is postulated to cause the strength decrease in the unsealed cured samples. These results show that water is a crucial component for the evolution of high strength three-dimensional cross-linked networks in geopolymers.

## 1. Introduction

The procedures and formulations that are employed to manufacture geopolymers are not standardized. Low-calcium fly ash, blast-furnace slag and metakaolin are the most widely used silicon and aluminium source material for producing geopolymers. Natural pozzolanic materials are also used to produce geopolymers [1,2]. The alkali hydroxides and sodium silicate are most frequently used as alkaline activators to generate a high pH and activate source materials. Many studies experimentally derive their own formulations and procedures for mixing and curing to achieve adequate workability and subsequent strength development of geopolymers. Many researchers consider bulk SiO_2_/Al_2_O_3_ and Na_2_O/Al_2_O_3_ or Na_2_O/SiO_2_ molar ratios to produce optimum mix proportions. On the other hand, it has been demonstrated that formulating the mixture based on amorphous composition of fly ash improves the mechanical performance of fly ash-based geopolymers [3,4,5]. Aside from variation in geopolymer formulation there are numerous curing schemes incorporating changes in temperature, and time, including recently-reported intermittent curing [6]. A wide range of curing temperatures and durations have been adopted; ranging from room temperature up to 90 °C, sometimes even over 100 °C for accelerated curing and wet curing schemes. Some researchers start with ambient temperature curing and then follow this with heat curing. The wide range of formulations and curing regimes conducted in research reflects the complexity of optimizing the wide range of parameters necessary to ensure high performance geopolymers are produced. 

How geopolymers are cured or aged is important in practical applications such as the precast concrete industry. Ambient cured geopolymers continue to increase in strength with age in the initial few weeks [7]. Curing geopolymers in sealed moulds is generally recommended to avoid loss of water. Water is consumed during dissolution of aluminosilicate source material that occurs in the formation of geopolymers [8]. The water is then released during hydrolysis, polymerization and condensation [8]. There is an opinion that the water in a geopolymer concrete mixture plays no role in the chemical reaction and merely provides workability to the mixture [9], but a few studies have discussed the important effects of water retention on the geopolymerization process and properties of metakaolin-based and fly ash-based geopolymers [10,11,12,13,14]. 

Fundamentally, the performance of a geopolymer product depends on the formulation, mixing procedure and curing scheme. In this work geopolymers are synthesized using the same approach proposed by Williams and van Riessen (2011) [4] under different curing conditions in order to investigate the role of water during curing. The aim of this study is to identify the effect of water retention on strength of sealed versus unsealed curing of geopolymers and add to information already available in the literature [13,14]. In addition, a new curing scheme for achieving high strength fly ash-based geopolymers is recommended.

## 2. Experimental Procedure

Cement grade, cleaned fly ash produced by Sampyo Cement Corporation was milled for 60 min to reduce particle size in a vibratory ball mill (MB-1, Dalton Co., Ltd., Tokyo, Japan). Four kilograms of 10 mm diameter steel balls was added to 250 g fly ash in a shell. The particle size distribution of the as-received and milled fly ash was measured by means of a particle size analyser (Mastersizer 2000, Malvern Co., Malvern, UK). The ash was milled to maximise the amount of amorphous aluminosilicate available to the alkali activator; this in turn increases the mechanical strength of the resultant geopolymers. The milling also ensures that the measured amorphous Si:Al in the precursor is close to, or the same, as that involved in the dissolution process thus optimising our mixing ratios.

The activator was a combination of sodium hydroxide (Junsei Chemical, Tokyo, Japan) and sodium silicate solution (SiO_2_ 32.1 wt %, Na_2_O 12.5 wt %, Youngil, Korea). The mix proportion was calculated to achieve target Si/Al and Na/Al ratios in the geopolymers of 3.5 and 1.0, respectively with water content of 17 wt %. The pH of the combined alkaline solution was 11.15. Mixing was done with a planetary centrifugal mixer (ARE250, Thinky Co., Tokyo, Japan) for 5 min at 1000 rpm in mixing mode and for 30 s at 2100 rpm in defoaming mode. The fresh mix was poured into cylindrical moulds with a diameter of 2.9 cm and height of 5.8 cm. Compressive strength was measured by using the MTS 815 Universal Testing Machine with a loading rate of 5.5 × 10^−3^ mm/s (MTS Systems Corp., Eden Prairie, MN, USA). Samples were tested 1, 7, 10, 14 and 28 days after synthesis and the compressive strength values are the average of the results from four samples.

Quantitative X-ray diffraction phase analysis was conducted using the DIFFRACPLUS and TOPAS 4.2 (Bruker-AXS GmbH, Karlsruhe, Germany) software. Calcium fluoride (CaF_2_, 99.985%, Alfa) was used as an internal standard to comprise 10.0000% of the sample weight. The mixture of fly ash and calcium fluoride was ground in a micronizer mill (McCrone, Westmont, IL, USA) for 5 min. X-ray diffraction (XRD) patterns were obtained from a powder X-ray diffractometer with Bragg-Brentano geometry using copper Kα radiation (D8 Advance, Bruker-AXS, Karlsruhe, Germany) over the 2-theta range 5°~80° with a step size 0.01° for 1 s/step. The chemical composition of fly ash was analysed by using X-ray fluorescence spectroscopy (Shimadzu Sequential XRF-1800, Shimadzu Scientific Instruments, Kyoto, Japan). Thermogravimetric analysis was performed in air from room temperature up to 900 °C with a heating rate of 5 °C/min (DTG-60H, Shimadzu Scientific Instruments, Kyoto, Japan).

All samples were sealed in moulds and placed in an oven at 70 °C for 1 day and then returned to ambient temperature. The “A” series specimens were kept in sealed moulds until testing while the “B” series specimens were demoulded and left unsealed until testing (Table 1). It should be noted that these samples were left unsealed so we could assess how the mechanical strength of geopolymers is influenced when they are exposed to a naturally-ventilated environment.

The unsealed BD specimens were aged until the daily weight loss of specimens decreased to less than 0.1%. The BD specimens were weighed from the 3rd to 12th October in the laboratory in 2015. The room temperature and humidity in the laboratory were recorded during this period and the compressive strength was measured on the last day. The compressive strength of the sealed AD specimens was obtained on the same day as the BD specimens were measured.

Specific surface area and Barrett-Joyner-Halenda (BJH) pore size distribution were determined by means of the Brunauer-Emmett-Teller (BET) method (TriStar 3000, Micromertics, Norcross, GA, USA).

The ^29^Si Magic-Angle Spinning Nuclear Magnetic Resonance (MAS NMR) spectra were obtained using a 500 MHz Solid State FT-NMR (Varian Unity INOVA) spectrometer, operating at 99.3152 MHz. A spinning speed of 20 kHz was used with a delay time of 1 s and 2000 accumulations were used to collect spectra. The chemical shifts were measured with respect to zero reference from tetramethylsiane (TMS).

## 3. Results and Discussion

### 3.1. Characteristics of Fly Ash

**Figure 1:**
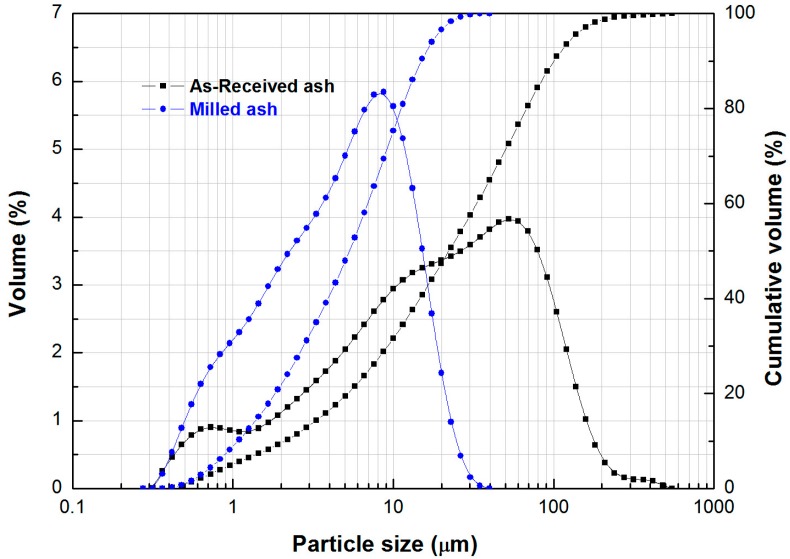
Particle size distribution for as-received ash and milled ash analysed by means of a laser diffractometer. Top cut (D97) particle size was reduced from 156.2 to 20.5 μm.

“Top cut (D97)” particle size was reduced from 156.2 to 20.5 μm by ball milling (Figure 1). The as-received fly ash has a mean particle size of 22.3 μm which decreased to 5.3 μm by milling (Figure 1). The as-received ash has three distinct particles size modes centred at about 0.5, 10 and 55 μm diameter. Milling eliminated the nodes, substantially narrowing the particle size distribution of the ash (Figure 1). SEM images of the milled ash are not presented but intact spherical particles were still observed.

The chemical composition of the ash as determined by XRF is shown in Table 2. The ash mainly consists of silica (53.49 wt %), alumina (21.54 wt %), iron oxides (8.15 wt %) and calcium oxide (5.77 wt %). The Rietveld quantitative phase analysis revealed that the major crystalline phases were mullite (15.0 wt %), quartz (14.9 wt %) and maghemite C (3.2 wt %). The amorphous fraction was calculated to be 66.9 wt %. The Si/Al ratio of the reactive component of the ash was determined to be 2.83. The reactive component of the ash was used to calculate the amount of silicate solution and NaOH required to achieve the targeted Si/Al ratio of 3.5 and Na/Al ratio of 1.0 in the geopolymers.

### 3.2. Impact of Unsealed Curing on Physical Properties of Geopolymers

The room temperature ranged between 20 and 25 °C while humidity fluctuated between 30% and 50% during the weight loss measurements of the unsealed geopolymers. BD specimens lost 4 wt % of their initial weight during the first three days; over the next four days the rate of weight loss decreased until stabilizing with an average total loss of 5.54 wt % by the 10th day.

**Figure 2:**
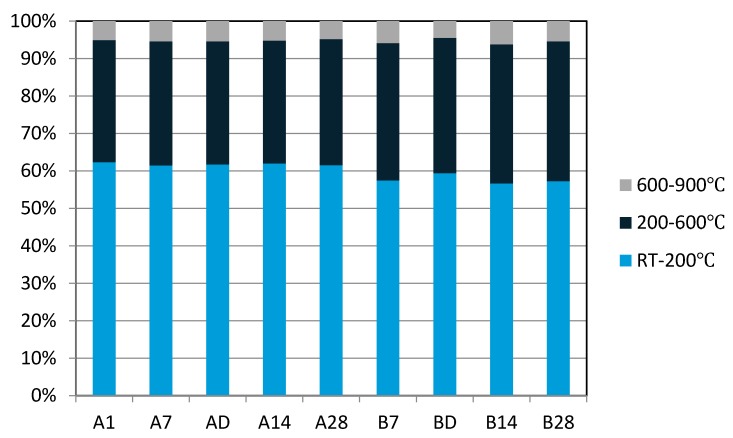
Percentage weight loss with temperatures ranging from room temperature to 900 °C.

**Figure 3:**
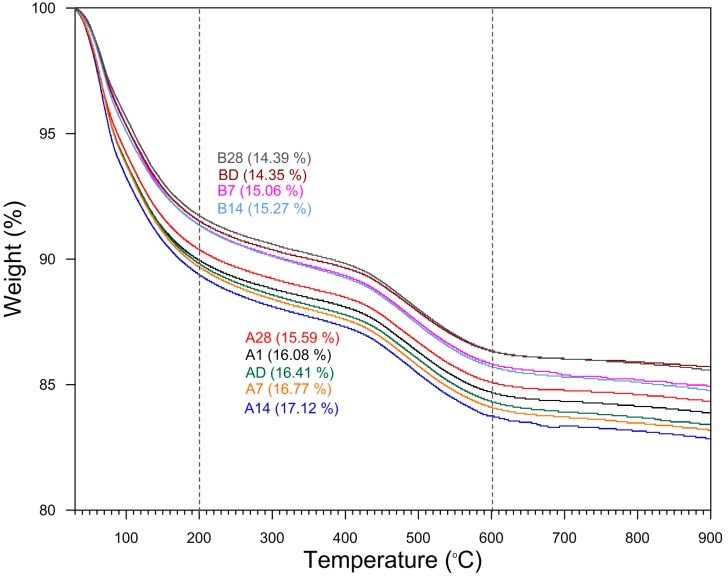
Thermogravimetric curves for the sealed “A” and the unsealed “B” series geopolymer specimens. The sealed “A” series specimens lost relatively more moisture at low temperatures and the weight loss at high temperatures was reversed.

In thermogravimetric analysis, the weight loss from room temperature to 200 °C was higher in the sealed “A” series specimens than in the unsealed “B” series specimens (Figure 2 and Figure 3). The percentage weight loss measured over this temperature range represented about 61%~62% of the total weight loss in the sealed “A” series specimens, while it ranged from 57% to 59% in the unsealed “B” series specimens (Figure 2). However, the weight loss in the range from 200 to 600 °C was reversed; being higher in the unsealed “B” series specimens (36%~37%) than in the sealed “A” series specimens (32%~33%) (Figure 2). In other words, the sealed “A” series specimens lost relatively more moisture from room temperature to 200 °C and less moisture from 200 to 600 °C compared to the unsealed “B” series specimens. This observation is consistent with the unsealed “B” series samples having already lost some water to the atmosphere during aging so proportionally they have less water to lose during heating.

**Figure 4:**
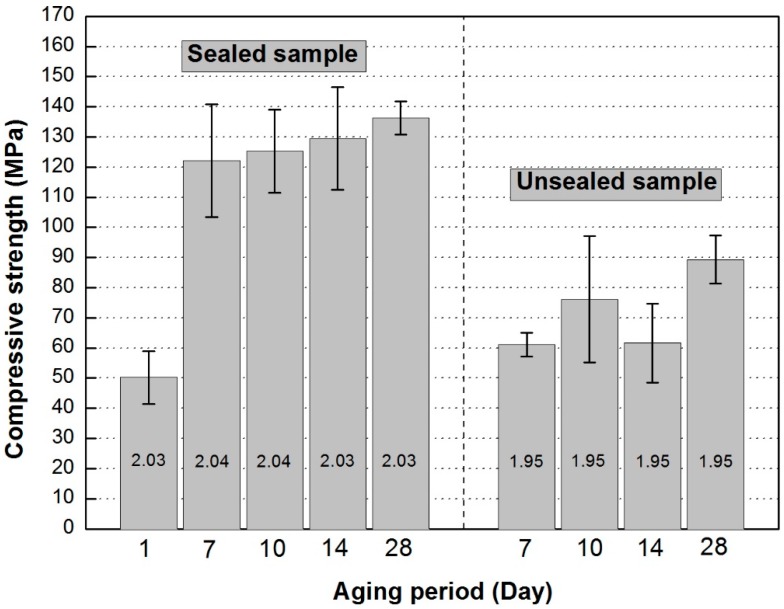
Compressive strength of geopolymers of the sealed “A” series specimens and the unsealed “B” series specimens. “A” series specimens in sealed moulds presented higher compressive strength at testing periods. Numbers in the bars are the apparent density (g/cm^3^).

The average compressive strength measured after finishing 1-day curing was about 50 MPa (Figure 4). After 7 days aging the average compressive strength of sealed geopolymers surpassed 120 MPa (Figure 4). The compressive strength showed a slight increase as the aging period increased in the “A” series samples. In the unsealed specimens the average compressive strength of geopolymers was lower, in the range 60~90 MPa with a slight increasing trend with increase in aging time. Clearly the compressive strength of the sealed specimens was significantly greater than that of the unsealed specimens in spite of the same mix proportion of geopolymers. Criado et al., (2010) measured strengths of sealed-cured samples of around three and half times more than unsealed-cured samples [14]. However, their curing regime was conducted at 85 °C for the entire time. They claim that for the unsealed-cured samples carbonation retards or halts the geopolymerization, yielding fairly low compressive strengths; in fact, they were able to measure the presence of bicarbonates by X-ray diffraction (XRD). In this study all specimens were sealed in an oven for the initial 24 h and the mix proportion was designed to achieve the targeted Na/Al ratio of 1.0. A very high compressive strength of the sealed geopolymers indicates that geopolymer reaction took place almost fully (Figure 4). In addition, the pH (minimum 10–10.5) of the pore solution of the carbonated geopolymer concrete is not significantly affected by carbon dioxide [15]. Consequently, carbonation is not thought to be the reason for lower strength of the unsealed geopolymers. It is worth noting that Criado et al., (2010) also observed the presence of zeolites in both sealed and unsealed samples using XRD [14]. 

What is so unusual for the sealed samples in this study is the dramatic strength increase after the initial 1-day curing at 70 °C. In many studies low calcium-geopolymers reach their ultimate strength if cured at elevated temperatures (70–90 °C) for about a day while ambient curing exhibits a gradual increase in strength with time [7,16,17,18,19,20,21]. Williams et al., (2011) conducted work on metakaolin-based geopolymers and concluded that a decrease in the amount of reacted metakaolin results in lower mechanical properties in the resultant geopolymer due to changes in matrix chemistry (Si/Al) and to a lesser extent on the amount of geopolymer binder [5]. Ascertaining changes in Si/Al in geopolymer and/or the amount of geopolymer accurately would go a long way to explaining our observations but these measurements are time consuming and difficult and outside the scope of the experiment. Nevertheless, it is hypothesized that the initial 1-day curing at 70 °C has facilitated sufficient dissolution of Si and Al, creating an environment where consolidation of the monomers and subsequently combination of these monomers to form a 3D structure is able to occur at ambient temperature while water is present. 

The apparent density of the sealed specimens was 2.03~2.04 g/cm^3^, which is higher than the 1.95 g/cm^3^ of the unsealed specimens. Figure 2 shows that the unsealed “B” series samples have lost an average of about 4% water to the atmosphere before heating commenced. The loss of water to the atmosphere during curing for the unsealed samples may have resulted in fine surface cracking that has resulted in strength loss (Figure 4). If a sample loses water but does not shrink, then it is clear its density will drop while at the same time creating internal stresses potentially weakening the sample. 

**Figure 5:**
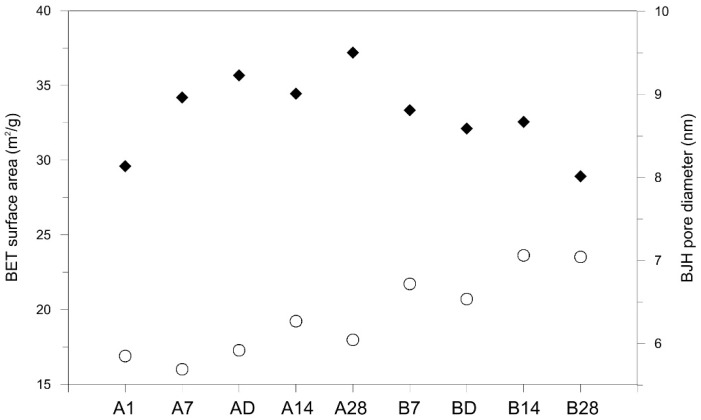
BET surface area (♦) and BJH desorption average pore diameter (◦) of geopolymers. The sealed specimens presented higher BET surface area with smaller pore diameter compared with the unsealed specimens.

The BET specific surface area and BJH pore size distribution of geopolymers showed substantial difference between the sealed and unsealed geopolymers (Figure 5). The sealed specimens had larger surface area, about 34~37 m^2^/g, but smaller pore diameter, about 5.7~6.3 nm, for A7, AD, A14 and A28. The unsealed “B” series specimens had smaller surface area, about 29~33 m^2^/g, with larger pore diameter, about 6.5~7.1 nm (Figure 5). There is a clear trend of increasing surface area with aging time for the sealed samples. This in conjunction with increasing strength with aging time suggests continued evolution of the geopolymer paste for the sealed samples. There is a marginal increase in pore diameter with increase in aging time for the sealed samples that is inconsistent with the related increase in surface area but as the trend is weak this will not be pursued further. For the unsealed samples the surface area decreases with aging time and this is concomitant with an increase in pore diameter. One explanation for this is that the loss of water during aging is promoting consolidation of pores (increase in size) as dehydration forces come into play. 

In their polarization and fluorescence microscopy study, Valcke et al., (2012) insist that even a geopolymer with a high strength has a microstructure consisting of a lot of air voids and shrinkage microcracks in between regions of dense geopolymer paste [22]. It is clear that the loss of water in the unsealed samples is the main contributor to lower strength evolution; a component of this strength loss is presumably related to dehydration microcracking. Cracks were not distinguished in visual inspection, but it can be assumed that invisible fine cracks created during rapid dehydration in the unsealed samples degrade strength performance. The larger pores of the unsealed samples may also be the result of more water escaping from the interior of the sample to the atmosphere (Figure 5). Porosity was not explicitly determined for the sealed and unsealed samples, but higher porosity of the unsealed samples could be the possible reason for lower strength if based on the lower apparent density. The strengths of hardened cement pastes and concretes, as well as that of any brittle material, decreases rapidly with an increase in porosity [23]. The reason for the significant strength reduction is a combination of three factors; (1) not only do the pores decrease the quantity of solid materials but (2) they also reduce the number of bonds, and most important; (3) they act as stress concentrators [24].

### 3.3. Role of Water during Sealed Aging in Geopolymerization

Higher compressive strengths in the sealed samples (Figure 4) indicates that geopolymerization continues in the hardened paste in addition to protection from dehydration cracking. In research on structural evolution during geopolymerization conducted by means of electron paramagnetic resonance, water is observed to be consumed during the hydrolysis/dissolution of metakaolin and then regenerated by polycondensation reactions and is then enclosed in the pores of the hardened geopolymer which is 15 days old [11]. During geopolymerization the newly formed oligomers or nanoaggregates interact to form the geopolymer network over time [11]. Structural evolution is also well-known in hardened cement. Short- and medium-range surface forces mediated by partially or totally hydrated calcium ions are the essential components of cement strength, with additional contributions from van der Waals and capillary forces [25]. In a conceptual model for geopolymerization proposed by Duxson et al., (2007) it is emphasized that each stage of geopolymerization (dissolution, gelation, polymerization and hardening) does not progress simultaneously because the overall process is heterogeneous [8]. Therefore, even if the geopolymer paste is already hardened, the role of water is crucial in further strength development of geopolymers. 

The empirical formula of geopolymer is known as

M*_n_*[(-SiO_2_)*_z_*-AlO_2_]*_n_*·wH_2_O where M is an alkali metal cation such as Na^+^ or K^+^, n is the degree of polycondensation and *z* is 1, 2 or 3 [26]. Water is included as an integral part of the geopolymer structure but it is not yet fully understood how water contributes to the strength development of geopolymers. In a study of metakaolin-based geopolymers high water content during late stages of geopolymerization is presumed to accelerate the polycondensation rate, which is based on heat evolution of metakaolin mixed with NaOH [27].

**Figure 6:**
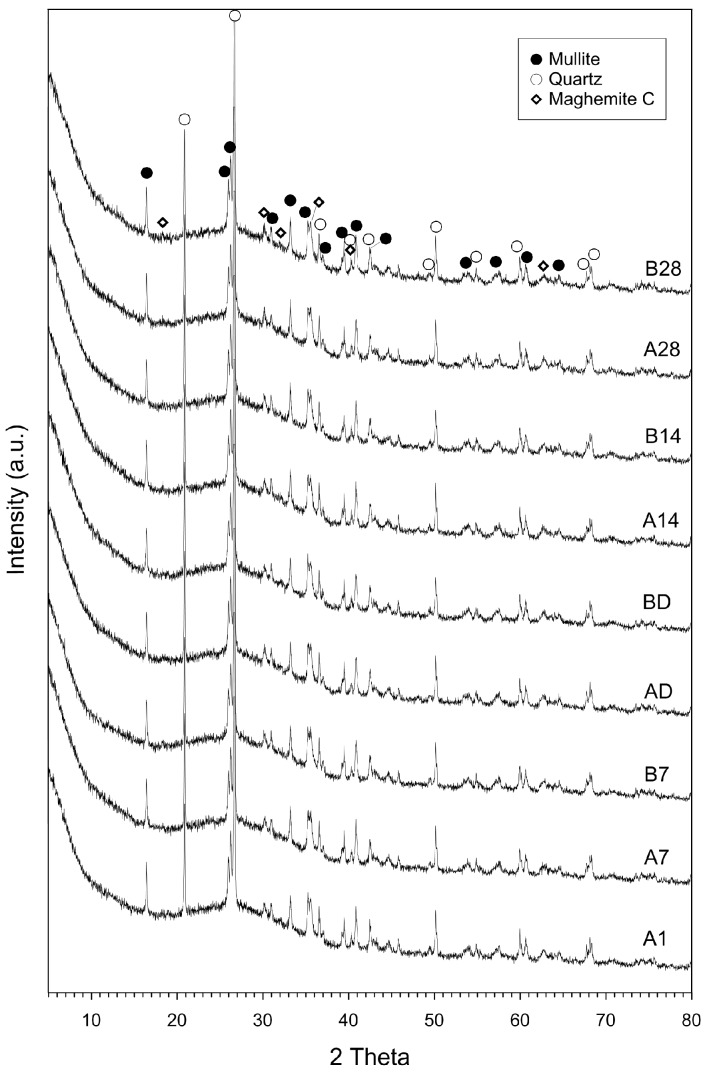
X-ray diffraction patterns of geopolymers described in Table 1. XRD patterns do not show any differences that could explain the substantial strength difference between the “A” and “B” series specimens.

**Figure 7:**
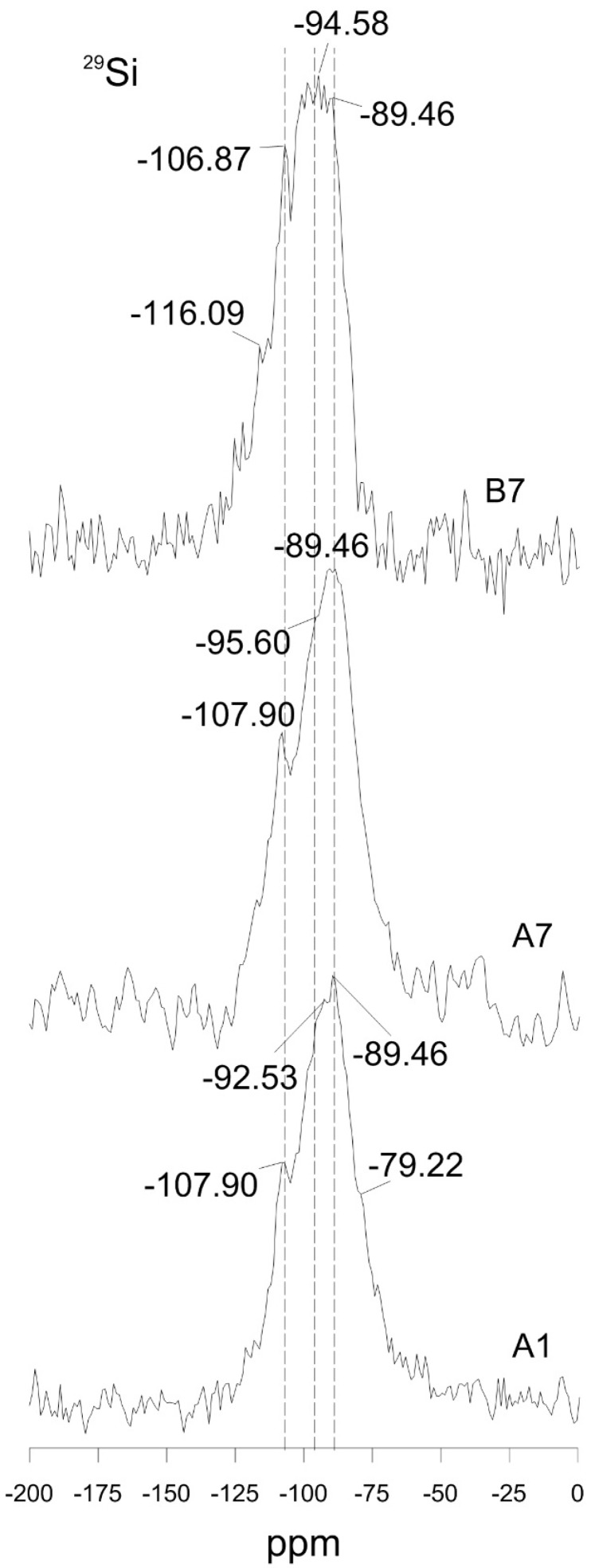
NMR spectra of samples A1, A7 and B7.

X-ray diffraction patterns yielded no discernible difference for geopolymers with different mechanical performance. (Figure 6). However, sealing the moulds probably resulted in differences in short-range ordering and eventually development of higher strength in A7 (Figure 4 and Figure 7). The ^29^Si MAS NMR spectrum of B7 exhibited peaks at lower frequency and relatively more asymmetry than A1 and A7 that both showed similarity in peak shape and resonance frequency (Figure 7). Increasing the nominal Si/Al ratio of geopolymers results in the ^29^Si MAS NMR resonance peak shifting to more negative values [28]. A geopolymer structure may consist of various Q unit types of connected SiO_4_ and AlO_4_ tetrahedra depending on chemical composition [29]. The ^29^Si NMR spectra of A1 and A7 taken after 1 and 7 days of aging showed similarities in chemical shift and their broad resonance lines indicating sheet sites, Q^3^ (4Al) and Q^3^ (0Al) units, were observed at around −89 and −95 ppm [30], respectively (Figure 7). In addition, A1 and A7 show another well-resolved peak at −107.9 ppm protruding from a dominant resonance at −89 ppm and indicating a three-dimensional cross-linked site, Q^4^ [30]. On the other hand, the dominant resonance of B7 aged for 7 days (unsealed) showed a different appearance from A1 and A7; fine structure in peaks at −89.46, 92.54, 94.58, 98.68, 100.73, and 106.87 ppm with a small peak at 116.09 ppm, which indicates a wider range of Q^3^ environments corresponding to cross-linked silicate bonds than in A1 and A7. Signals at 100.73, 106.87 and 116.09 ppm are probably from the unreacted vitreous phase [14] or unreacted silicate oligomers that are not bound to the gel [28], indicating that loss of water contributes to slower geopolymerization and lower mechanical performance of B7.

## 4. Conclusions

For the fly ash-based geopolymers used in this study significantly higher strength was achieved by leaving the samples sealed during aging. The average compressive strength measured after 1 day of curing was about 50 MPa. After 7 day aging the average compressive strength of geopolymers reached about 120~135 MPa when they were aged in the airtight sealed moulds. In the unsealed specimens the average compressive strength of geopolymers was lower, in the range of 60~90 MPa with a somewhat increasing trend as the aging period increased. For the unsealed samples further structural evolution is blocked by dehydration resulting in hindering continuous reorganization of the polycondensation processes and subsequent lower strength relative to sealed samples. Dehydration microcracking is also postulated to cause strength decrease in the unsealed samples. These results show how crucial it is to seal geopolymers during aging as the presence of water enables on going geopolymerisation resulting in higher density, smaller pores and greater compressive strength. 

For the specific fly ash used and experimental conditions adopted it would appear that a 1-day sealed-cure at 70 °C followed by further sealed-aging at ambient temperature enables impressive strength gains to be realized, which is ideal for precast products. 

## Figures and Tables

**Table 1:** Description of geopolymer specimens and their curing regime.

Specimen	Strength Measurement Time
A1	After 1 d * curing at 70 °C
A7	1 d curing at 70 °C + 6 d aging sealed at ambient temperature
AD ^1^	1 d curing at 70 °C + 9 d aging sealed at ambient temperature
A14	1 d curing at 70 °C + 13 d aging sealed at ambient temperature
A28	1 d curing at 70 °C + 27 d aging sealed at ambient temperature
B7	1 d curing at 70 °C + 6 d aging unsealed at ambient temperature
BD ^1^	1 d curing at 70 °C + 9 d aging unsealed at ambient temperature
B14	1 d curing at 70 °C + 13 d aging unsealed at ambient temperature
B28	1 d curing at 70 °C + 27 d aging unsealed at ambient temperature

* 1 d means 1 day; ^1^ Samples AD and BD are cured for a total of 10 days, with AD sealed and BD unsealed.

**Table 2:** Chemical composition of the fly ash used in this study (wt %). LOI = loss on ignition.

SiO_2_	Al_2_O_3_	Fe_2_O_3_	CaO	MgO	K_2_O	Na_2_O	TiO_2_	MnO	P_2_O_5_	LOI
53.49	21.54	8.15	5.77	2.02	1.30	0.90	1.17	0.09	1.00	4.13
